# Indicators and measurement tools for health system integration: a knowledge synthesis protocol

**DOI:** 10.1186/s13643-015-0090-7

**Published:** 2015-07-29

**Authors:** Nelly D. Oelke, Esther Suter, Maria Alice Dias da Silva Lima, Cheryl Van Vliet-Brown

**Affiliations:** School of Nursing, University of British Columbia, Okanagan 3333 University Way, Kelowna, British Columbia V1V 1V7 Canada; Workforce Research & Evaluation, Alberta Health Services, 10301 Southport Lane SW, Calgary, Alberta T2W 1S7 Canada; Escola de Enfermagem, Universidade Federal do Rio Grande do Sul, Rua São Manoel, 963, Porto Alegre, Brazil

**Keywords:** Delphi, Focus groups, Health systems, Integration, Knowledge synthesis, Systematic review

## Abstract

**Background:**

Health system integration is a key component of health system reform with the goal of improving outcomes for patients, providers, and the health system. Although health systems continue to strive for better integration, current delivery of health services continues to be fragmented. A key gap in the literature is the lack of information on what successful integration looks like and how to measure achievement towards an integrated system. This multi-site study protocol builds on a prior knowledge synthesis completed by two of the primary investigators which identified 10 key principles that collectively support health system integration. The aim is to answer two research questions: What are appropriate indicators for each of the 10 key integration principles developed in our previous knowledge synthesis and what measurement tools are used to measure these indicators? To enhance generalizability of the findings, a partnership between Canada and Brazil was created as health system integration is a priority in both countries and they share similar contexts.

**Methods/design:**

This knowledge synthesis will follow an iterative scoping review process with emerging information from knowledge-user engagement leading to the refinement of research questions and study selection. This paper describes the methods for each phase of the study. Research questions were developed with stakeholder input. Indicator identification and prioritization will utilize a modified Delphi method and patient/user focus groups. Based on priority indicators, a search of the literature will be completed and studies screened for inclusion. Quality appraisal of relevant studies will be completed prior to data extraction. Results will be used to develop recommendations and key messages to be presented through integrated and end-of-grant knowledge translation strategies with researchers and knowledge-users from the three jurisdictions.

**Discussion:**

This project will directly benefit policy and decision-makers by providing an easy accessible set of indicators and tools to measure health system integration across different contexts and cultures. Being able to evaluate the success of integration strategies and initiatives will lead to better health system design and improved health outcomes for patients.

## Background

Integration in health care is a key component of health care reform [1]. Given our aging population and higher rates of chronic disease, there has been a shift from acute, episodic care to a greater focus on integrated care across the continuum [1, 2]. However, delivery of health care services continues to be very fragmented [3]. Two of the authors (NO/ES) previously conducted a Canadian Institute of Health Research-funded knowledge synthesis [4] focused on models for health system integration. The results showed there was no definitive health system integration model appropriate for all organizations given the complexity of health care service delivery. Despite the lack of a definitive model, 10 key principles were identified that collectively support health system integration. The principles include (1) comprehensive services, (2) patient focus, (3) geographic coverage and rostering, (4) standardized care delivery through interprofessional teams, (5) performance management, (6) information technology, (7) organizational culture and leadership, (8) physician integration, (9) governance structure, and (10) financial management [5].

Integration initiatives are being implemented at all levels of the health care system [1]. Health system integration has enduring relevance provincially, nationally, and internationally. Many jurisdictions continue to grapple with the development of integration strategies and how to measure integration. For example, in British Columbia (BC), the 10 key principles have been used by the BC Ministry of Health, Interior Health and the Michael Smith Foundation for Health Research to guide health care reform and evaluation of integrated community health services. In Porto Alegre, Brazil, a working group was established to create networks to increase primary health care integration [6]. Internationally, the World Health Organization (WHO) hosted a technical meeting, “WHO Strategy on People-Centered and Integrated Health Services” to discuss and review indices and measures [7].

Health systems are consistently striving to deliver integrated health services as integrated models have the potential to positively impact patient, provider, and system outcomes. More specifically, integration has the potential to improve quality of care [1, 8] and decrease utilization of resources [2, 8]. Despite health care organizations’ efforts to achieve integration, there is little information on what successful integration looks like and how to measure achievement towards an integrated system [9]. An important gap that emerged in the prior knowledge synthesis was the lack of indicators and tools to measure integration. Some organizations have developed balanced scorecards around particular integration components [10]. Others have used the Clinical Microsystem Assessment Tool [11], which allows a snapshot of where an organization lies along a continuum of integration. Strandberg-Larsen and Krasnik [1] argue that “methods to measure integrated health care delivery are clearly emerging” (p. 4); however, the few tools that exist are not easy to find as literature on integration is dispersed. Likewise, it is unclear if the tools that currently exist cover all 10 principles of integration as identified in our previous knowledge synthesis and there is no inventory of indicators and tools that can be accessed by decision-makers to develop evaluation and performance monitoring plans. This hampers our collective ability to monitor the effectiveness of integration strategies and limits our ability to improve health system integration [12].

There are five reasons why this knowledge synthesis is significant and urgently needed: (1) Current systems continue to be fragmented. (2) Many health systems include goals focusing on integrated health care. (3) Most health systems are implementing integration initiatives designed to improve quality of care and efficiency while reducing costs and resource utilization. (4) There is a lack of understanding by health systems of their achievement towards integrated health care. (5) There is a lack of tools available to measure health system integration.

This knowledge synthesis will address gaps in the measurement of integration in health systems. Our research questions include the following: (1) What are appropriate indicators for each of the 10 key integration principles developed in our previous knowledge synthesis? And (2) what measurement tools are used to measure these indicators? This project will directly benefit policy and decision-makers by providing an easily accessible set of indicators and tools to measure health system integration across different contexts and cultures. Being able to evaluate the success of integration strategies and initiatives will lead to better health system design and improved health outcomes for patients. To enhance the global applicability of the proposed work, we have developed a partnership between Brazil and Canada. This partnership was specifically chosen as both countries have publicly funded health systems, comparable funding priorities, and similar geography of large urban centres and rural communities. Furthermore, health system integration is a priority in both countries.

## Methods/design

This knowledge synthesis will follow processes for scoping reviews recommended by Levac et al. [13]. They stress the need for an iterative process rather than a linear process with emerging information leading to refinement of research questions and study selection. The Levac et al. [13] framework was selected based on its applicability and relevance given the policy context of the questions, the need for an iterative process, and knowledge-user engagement. This methodological framework outlines six stages for rigorous scoping reviews: (1) identifying the research question; (2) identifying relevant studies; (3) selecting studies; (4) charting the data; (5) collating, summarizing, and reporting results; and (6) consulting [13]. For this knowledge synthesis, an additional stage termed identifying indicators was added following identifying the research question. As this review does not address the effects of interventions and/or strategies to prevent, diagnose, treat, and/or monitor health conditions, for which there is a health-related outcome, it is ineligible for PROSPERO registration.

### Identifying the research question

Identifying the research question requires consideration of the scope of inquiry and the purpose of the review [13]. The scope of inquiry for this review is to identify and validate relevant integration indicators associated with the 10 key principles and measurement tools to monitor progress towards integrated health systems. The purpose is to enable evaluation of the success of integration strategies and initiatives, which will lead to better health system design and improved health outcomes for patients. Knowledge-users were consulted on the issues of integrated health systems and the focus of our knowledge synthesis. Research questions were developed and shared with our knowledge-users who confirmed the importance of these questions. The research questions to be addressed by the scoping review include the following: What are appropriate indicators for each of the 10 key integration principles and what measurement tools are used to measure these indicators?

### Identifying indicators

As part of the knowledge synthesis and prior to conducting our systematic review on existing tools to measure health system integration, a better understanding of the indicators for successful integration is needed. Two approaches to prioritize and identify indicators will be utilized. A Delphi survey with key stakeholders will be used to identify appropriate indicators and priorities for the same. The second approach, focus groups with patients or users of the health system, will determine patient and user prioritization of the principles the indicators measure. Drawing on previous work and a scan of the literature, research team members generated a preliminary list of possible indicators for each of the 10 key principles (see Table 1 for sample indicators). Research team members including researchers and knowledge-users confirmed the preliminary list of indicators.

**Table 1:** Sample indicators for each key principle

Key principle	Description of the principle	Sample indicators
1. Comprehensive services across the care continuum	• Cooperation between health and social care organizations • Access to care continuum with multiple points of access • Emphasis on wellness, health promotion, and primary care	• Coordinated transitions in care across services [23] • Shared programs across sectors/services [24] • Third next available appointment [25] • Emergency department average LOS registration to discharge; registration to admission (QPSD 23) [26] • Measure wait time for referral to treatment by provider type (QPSD 20) [26] • Proportion of patients with health outcomes which are avoidable given the current state of medical knowledge and access to appropriate care [27] • Tobacco screening [28]
2. Patient focus	• Patient-centred philosophy; focusing on patients’ needs • Patient engagement and participation • Population-based needs for assessment; focus on defined population	• Involvement in care planning for chronic disease/complex care [29] • Evidence of a population-based needs assessment [30, 31]
3. Geographic coverage and rostering	• Maximize patient accessibility and minimize duplication of services • Roster: responsibility for identified population; right of patient to choose and exit	• Existence of primary care network structures (e.g. family health teams, primary care networks, GP Divisions, inner city primary health care clinics) [30]
4. Standardized care delivery through interprofessional teams	• Interprofessional teams across the continuum of care • Provider-developed, evidence-based care guidelines and protocols to enforce one standard of care, regardless of where patients are treated	• Team effectiveness [32] • Using a shared clinical pathway across care sectors (e.g. diabetes care, asthma care) [33]
5. Performance management	• Committed to quality of services, evaluation, and continuous care • Diagnosis, treatment, and care interventions linked to clinical outcomes	• Performance measurement indicators and tools are in place and being used regularly [34] • Clinical outcomes being measured [35]
6. Information systems	• State-of-the-art information systems to collect, track, and report activities • Efficient information systems that enhance communication and information flow across the continuum of care	• Shared information systems across care sectors [36, 37]
7. Organizational culture and leadership	• Organizational support with demonstration of commitment • Leaders with vision who are able to instil a strong, cohesive culture	• Extent to which organizational goals and objectives are aligned across care sectors [36]
8. Physician integration	• Physicians are the gateway to integrated health care delivery systems • Pivotal in the creation and maintenance of a single-point-of-entry or universal electronic patient record • Engage physicians in leading role, participation on Board to promote buy-in	• Physician integration within care teams and across care sectors [10, 36, 38] • Practitioner payment models that support integration [37]
9. Governance structure	• Strong, focused, diverse governance represented by a comprehensive membership from all stakeholder groups • Organizational structure that promotes coordination across settings and levels of care	• Existence of interagency agreements, service delivery team coalitions [39] • Governance model that includes representation of communities served [30] • Evidence of governance in monitoring and evaluation of health system [40]
10. Financial management	• Aligning service funding to ensure equitable funding distribution for different services or levels of services • Funding mechanisms must promote interprofessional teamwork and health promotion • Sufficient funding to ensure adequate resources for sustainable change	• Extent to which financial management is coordinated across care units and sectors [36]
11. Overall integration		• Degree of integration within the health system and across sectors [41, 42]

#### Delphi survey

A modified Delphi method will be used to obtain consensus from a panel of integration experts, policy and decision-makers, and providers on the most relevant indicators for each key integration principle. This approach allows expert perspectives and judgments to be collected without the need for face-to-face or virtual meetings, thus reducing costs and logistical details [14]. It also reduces socially desirable responding as ratings are anonymous to the rest of the panel [15]. Panel members include individuals from Canada and Brazil, as well as international experts on the topic area. The survey will be translated into Portuguese to enable key stakeholders from Brazil to participate.

For each round of the Delphi process, the panel members (*n* = 30–35) will receive a survey with a list of the principles and indicators by email. They will be asked to rate the fit and importance of each indicator to its respective key principle using a 5-point Likert-type scale. Within each of the key principles, participants will also be asked to prioritize the indicators. Results will be compiled and used to inform subsequent rounds of the survey [16, 17] until consensus is attained. The determination of consensus is open to interpretation; however, it generally falls between 70 and 80 % [15]. In this study, a consensus level of 75 % agreement has been selected. Indicators will be deleted when they are considered not relevant demonstrated by 75 % of participants rating at 4 or 5 on appropriateness and importance and rated lower than 3 or 4 for priority. An indicator will be accepted once 75 % consensus is obtained on all three ratings.

##### Round 1

Panel members will receive the survey with a complete list of preliminary indicators. They will be asked to add any relevant missing indicators. Results will be compiled and indicators suggested by the panel members added. We anticipate there may be a substantial number of additional indicators suggested in round 1. First, these will be themed by a research assistant and reviewed by the primary investigator (PI) coordinating the study. Those indicators that are essentially the same will be combined. Second, the list of existing indicators, themed indicators, and other additional indicators will be circulated to the research team. All team members will be asked to rate the appropriateness, importance, and priority as per the Delphi survey scale. They will also be asked to provide rationale for their ratings. These results will be compiled and analysed. A sub-committee of the research team including PIs from each jurisdiction and a co-investigator with expertise in Delphi processes will discuss these results and decide on the indicators to be included in the second version of the Delphi survey.

##### Round 2

The round 2 survey will be sent out to all participants who were invited to participate in the first round except those who indicated they were not able to or did not wish to participate. In this round, there will be no opportunity for the addition of new indicators. Participants will only be asked to rate the appropriateness, importance, and priority of each indicator. The survey will then be revised to only include those indicators that are relevant and those where there is no agreement.

##### Subsequent rounds

The revised survey will be sent out to all participants invited as per rounds 1 and 2. Round 3 and subsequent rounds will be analysed in the same manner as round 2. We anticipate three to four rounds will be needed to achieve consensus among the panel members. Each of the indicators will be assessed individually for agreement. If there is 75 % agreement on fit and importance, they will be included in the final list. If there is no 75 % agreement across Delphi participants for a specific indicator, they will not be included in the final list. We will aim to settle on two or three indicators per key principle. This final set of indicators will provide the foundation for the systematic literature review.

#### Focus groups

One patient or user focus group consisting of four to eight individuals will be held in each of the regions (BC and Alberta, Canada, and Rio Grande do Sul, Brazil) to gain an understanding of patient perspectives on the principles of integration. This methodology was selected as it allows for the expansion of knowledge through purposeful interaction of group members to generate contextually grounded opinions and beliefs about a topic [18]. Each focus group will be facilitated by a member of the research team who is familiar with the 10 key principles of integration using a set procedure and interview guide. A second team member will take field notes and audio record the session. Participants will be provided with a list of the 10 key principles of integration and their descriptions. The handout will also have a column where they will be asked to prioritize the principles at the end of the session with #1 being a high priority and #10 being the lowest priority. Priority ratings for the principles will be analysed using descriptive statistics. Focus group recordings and field notes will be transcribed verbatim and coded using NVivo10™ software. A standardized coding framework built on the principles and indicators will be developed for use by all teams coding the data. A research assistant/PI team will code and conduct a thematic analysis of the data in Brazil, and another such team will analyse the two focus groups completed in Canada. They will then hold a virtual meeting to discuss the similarities and differences in themes. Key sections of data from Brazil will be translated into English, and a final round of analysis of all focus group data will then be conducted. These results will create an in-depth understanding of their perspectives on integration and which principles patients and users see as important to an integrated health system. To our knowledge, there is little research on patients’ perceptions on integration. This unique perspective will influence recommendations for which indicators should be prioritized for implementation or further research.

### Identifying relevant studies

The research librarian team member will assist with identification of search terms together with a sub-committee of the research team including researchers, research librarian, and research assistants. The initial search will focus on search terms relating to each of the indicators and will be reduced by including terms relating to health system integration and tools/tool development. Search terms for each indicator will be reviewed by the sub-committee and sample searches conducted prior to the final searches being executed by our librarian. Material about health system integration and related indicators may also be found in sources outside the traditional research literature. The search strategy will encompass both the peer-reviewed and targeted grey literature published from 1995 to 2015. The search for relevant literature will include [19] Health Sciences, Education and Management/Business bibliographic databases (Medline including the Cochrane Library, EMBASE, PsycINFO, CINAHL, ABI Inform, and Business Source Premier), websites of relevant government agencies and research organizations (e.g. Institute for Health care Improvement), scanning reference lists of included studies, contacting key authors to identify additional papers focusing on measurement tools, Web of Science citation searching, and consulting with experts to highlight key papers.

### Selecting studies

Abstracts will be downloaded into ProQuest RefWorks™ bibliographic management software program, and duplicates will be removed. An initial set of inclusion and exclusion criteria have been developed and reviewed by the research team. Inclusion criteria include quantitative, qualitative, and mixed-methods study designs, published in English or Portuguese languages, and published within the last 20 years (1995–2015) when integration in health systems became a more common topic of discussion. Priority will be given to randomized control trials and other quantitative studies that specifically discuss the development or use of a tool. In instances where there are no tools in the quantitative literature for an indicator, qualitative studies looking at research to support tool development will be included. Articles will be excluded if they were published prior to 1995, are from non-health care settings, or are of a theoretical, editorial, or commentary nature. The same approach will be used for the identification of other sources (e.g. websites) where reports, papers, and abstracts focused on measurement tools related to indicators will be downloaded into bibliographic software.

All research team members involved in reviewing abstracts will pre-test the criteria using 20 randomly selected abstracts. This will allow establishment of inter-rater reliability. Criteria will be refined if needed, and pre-testing repeated until the mean inter-rater reliability kappa of all pairs is satisfactory (kappa >0.8). Subsequently, pairs of raters from the team will independently use the criteria to screen each abstract from the peer-reviewed literature for relevance. Disagreements will be resolved by a third reader. Full-text articles for abstracts meeting relevancy criteria will be retrieved. Similarly, abstracts or executive summaries of grey literature reports will then be rated by pairs, and relevant full-text reports will be retrieved.

Pairs of raters will then independently screen for inclusion of full-text articles and reports for review. As with the abstract screening process, criteria for full-text inclusion will be developed and tested. If disagreement occurs, a third reader will review the article in question. Integration is a broad term, and to ensure important articles are not missed, a fairly high number of abstracts and full-text articles will be screened. We anticipate only a smaller number of these articles will meet the relevancy criteria. The bibliographies of full-text studies meeting inclusion criteria will be scanned to identify additional articles of possible relevance which will then undergo the same selection process. Ratings and selection of Portuguese abstracts and articles will be conducted by research team members in Brazil and will follow the same procedures as outlined above.

#### Appraisal of study quality

A quality appraisal tool [20] was adapted, tested, and implemented successfully by one of our primary researchers in a previous knowledge synthesis [21]. All selected studies will be independently assessed by two reviewers using the tool prior to data extraction. This step will be critical to ensure selection of only high-quality studies discussing measurement of the indicator(s) in enough detail to enable replication.

### Charting the data

#### Data extraction

Peer-reviewed articles and grey literature reports considered relevant will be thematically grouped by indicator to facilitate extraction of information. Prior to the commencement of charting of the data, an extraction template will be developed. The extraction categories to be included are author, year of publication, country of publication, integration principle, indicator, study type, sample population including health care context, name and description of tool, components of the tool, and limitations. This data extraction template will be tested by the reviewers on a small set of articles to determine the usefulness of the categories and identify any gaps in the template. Furthermore, consistency across data extraction will be determined, and inconsistencies will be discussed and resolved prior to moving along to further data extraction. Data will be extracted by a single reviewer with systematic audits completed to ensure accuracy and quality of extracted data.

### Collating, summarizing, and reporting results

From the extraction template, a listing of relevant tools available for each of the indicators will be compiled including key components of the tool (e.g. validity and reliability testing, type of tool). From these summaries, a narrative analysis of the studies will be developed addressing overall strengths and limitations of the knowledge base, the quantity of studies/articles for each indicator, measures and methods used, the quality of existing measures, questions addressed, and evidence gaps. Qualitative thematic analysis [13] will be used where appropriate for this synthesis.

Draft reports will be reviewed by the project team. The revised report will be circulated to the full research team. They will be invited to assess whether the summarized information in preliminary form has captured the indicators of interest as well as impressions about the validity of conclusions. They will also be asked to highlight the findings most immediately useful, help develop recommendations and key messages, and make suggestions for further formatting and communication. Feedback from the research team will inform revisions to the report. The final systematic review report will include a single page of key messages and summary of the policy context of the review, a three-page executive summary, the full report with appendices, and one or two additional user-friendly communication tools as suggested by knowledge-users.

### Consultation

This knowledge synthesis uses an integrated knowledge translation (KT) approach [22]. Throughout the development of the proposal and initial implementation of the research, we have been working directly with knowledge-users (decision-makers and policy-makers) in all of the research processes. We have included knowledge-users from each jurisdiction on our research team. These integrated KT approaches will ensure the relevance of the research and facilitate the dissemination and uptake of research results.

An end-of-grant KT event will be held for knowledge dissemination and exchange with researchers and knowledge-users from the three jurisdictions (Alberta, BC, and Brazil). Knowledge-user team members will be critical in identifying about 50 provincial, national, and international stakeholders to participate. The objectives of the meeting are to present the results from the systematic review for stakeholder discussion and validation, discuss implications of results within local contexts and how they will be used by different stakeholders, and identify outstanding questions. The meeting will be hosted through a blended format using face-to-face and internet technology to allow for broad participation and to reduce costs. Stakeholders will all be connected through the internet (e.g. Web-ex). Similar to videoconferencing, we can simultaneously reach all stakeholders for an overall presentation online, break-out for small interactive sessions, and reconnect as a larger group. Our team has successfully hosted a number of these distributed events with close to 100 participants. The mix between face-to-face and internet participation is a cost-effective way to enable networking amongst partners and other key stakeholders while at the same time creating synergies across the jurisdictions. All participants will receive a copy of the systematic review and a written report of the event proceedings.

### Ethical considerations

The research protocol was submitted to Ethics Boards at all three hosting sites: the University of British Columbia Okanagan Behavioural Research Ethics Board, University of Calgary Conjoint Health Research Ethics Board and Research Services Office, and Universidade Federal do Rio Grande do Sul Research Ethics Committee. Ethics approval has been received from all three sites. Participants in the Delphi study and focus group participants will be presented with a specific consent form outlining the research objectives, research implications, and measures to ensure confidentiality along with a list of the research team members and their contact information. They will be informed participation in the study is voluntary. Delphi study participants will be informed their consent is implied by electronic submission of the completed survey.

### Project status

At the time of submission of this paper, the second round of the Delphi process has been started. Focus groups in BC and Brazil have been completed.

## Discussion

The outputs of this knowledge synthesis are a list of indicators reflective of health system integration as established through panel consensus, a collection of detailed measurement tools for capturing each of the indicators, and a final report outlining the advantages and challenges with each indicator and measurement tool and its application for evaluating integration. This study will help stakeholders and policy-makers working in various jurisdictions on health system integration to measure the success of different strategies through appropriate indicators and tools. This will ultimately lead to better design of health care systems and better health outcomes.

A number of potential challenges could affect progress on this knowledge synthesis. First, given the nature of the concepts under study, a substantial number of potential indicators may be generated for consideration. Use of the modified Delphi technique will mitigate this issue, as panel members will be iteratively choosing indicators considered most important to the research questions. This will ensure a focus on only those indicators that are measurable, relevant, and meaningful. Second, the literature searches may result in a vast quantity of literature to examine. Past team experience with knowledge syntheses has facilitated the development of an efficient method of screening abstracts and rating full-text articles that allows for rapid movement through the preliminary stages and focus on extraction of relevant information. Third, the team has also developed effective strategies to conduct international research. Established working relationships currently exist with the Brazilian university. Language issues will be mitigated as all research team members are fluent in the English language.

## Abbreviations

BC: British Columbia
KT: knowledge translation
PI: primary investigator
WHO: World Health Organization
